# Anharmonicity of Plasmons in Metallic Nanostructures Useful for Metallization of Solar Cells

**DOI:** 10.3390/ma16103762

**Published:** 2023-05-16

**Authors:** Zofia Krzemińska, Witold A. Jacak

**Affiliations:** Department of Quantum Technologies, Wrocław University of Science and Technology, Wyb. Wyspiańskiego 27, 50-370 Wrocław, Poland

**Keywords:** metallic nanoparticles, surface plasmons, anharmonicity of oscillations, damping of plasmons

## Abstract

Metallic nanoparticles are frequently applied to enhance the efficiency of photovoltaic cells via the plasmonic effect, and they play this role due to the unusual ability of plasmons to transmit energy. The absorption and emission of plasmons, dual in the sense of quantum transitions, in metallic nanoparticles are especially high at the nanoscale of metal confinement, so these particles are almost perfect transmitters of incident photon energy. We show that these unusual properties of plasmons at the nanoscale are linked to the extreme deviation of plasmon oscillations from the conventional harmonic oscillations. In particular, the large damping of plasmons does not terminate their oscillations, even if, for a harmonic oscillator, they result in an overdamped regime.

## 1. Introduction

One of the most promising methods to increase the efficiency of solar cells is to apply metallic nano-components and take advantage of the mediation of the surface plasmons in metallic nanoparticles in the absorption of incident sunlight. This effect is especially noticeable in conventional p-n junction solar cells, such as Si-based cells or CIGS, where a dilute surface semiconductor covered with metallic nanoparticles (Au, Ag or Cu) with a diameter of 10–100 nm and surface concentration of only 10^8–9^ /cm^2^ can increase the photocurrent in the photodiode setup by a factor exceeding even 2 [1,2,3,4,5,6,7,8,9,10], leading to the surpassing of the Shockley–Queisser efficiency limit [11]. The related increase in the final efficiency of metallized solar cells is lower because not only photo-absorption contributes to the gain in a cell, but it can still achieve up to several dozen percent for the relative increase in the overall cell efficiency, as reviewed in [12]. The drawback of the application of metallic components is the low durability of surface coverings (some additional protective coatings should be applied, which, however, complicates the technology of cell production and increases costs). In the case of chemical solar cells, metallic nanoparticles can be incorporated into cell layers, and indeed, for hybrid chemical perovskite solar cells, gold or silver nanoparticles can be admixed with the liquid precursors and positioned in a controlled manner within the multilayered cell structure without perturbing their morphologies [13,14,15]. The record for the efficiency increase in perovskite solar cells due to metallization is 40% for the relative U-I overall gain [14]. Nevertheless, in perovskite cells, the increase in photon absorption is not the dominant plasmonic effect responsible for such large efficiency growth. This is due to the different operation scheme of perovskite cells in comparison to p-n junction cells. In the latter, the excitons induced by plasmons in the depletion region are instantly dissociated into separated electrons and holes by a high p-n junction voltage (in Si, of the order of 1 V, which is ca. 10 times higher than the binding energy of excitons, which does not exceed 100 meV). In perovskite cells (and in other chemical cells), no p-n junctions exist, and the dissociation of excitons occurs at the interface between the perovskite layer and electron (or hole, depending on the cell architecture) transport layer. The mutual shift of the conduction (or valence for holes) band edges in two adjacent materials decouples e-h pairs at the interface, and electrons (holes) are captured in the transport layer, resulting in a photocurrent. The lower the binding energy of excitons, the more efficient this process. It has been demonstrated [16] that, by applying metallic nanoparticles, one can reduce the binding energy of excitons induced by plasmons by even 50%, which explains the observation of a 40% efficiency increase [14] without an absorption increase [17]. This reveals the additional role of plasmons besides strengthening the photo-effect (note that a reduction in exciton binding is unimportant in p-n junction cells, because the junction voltage is one order of magnitude larger than the exciton binding energy, and the reduction in the latter does not have any effect).

In all these plasmon-related photovoltaic phenomena, the specific properties of surface plasmons in metallic nanoparticles are of primary importance. The conventional classical approach to plasmons entails determining the solution of Maxwell equations for a boundary problem of metallic nanoparticles (in a similar way to solving the Fresnel equation), which allows for the identification of surface plasmon resonance in metallic particles, which are then regarded as oscillators of localized dipoles (as the most important mode for surface plasmons at the nanoscale is the dipole mode with a corresponding wavelength (for Au, Ag or Cu) ca. one order larger than the confinement scale). Nevertheless, the simplified treatment of surface plasmons in metallic nanoparticles as ordinary dipole harmonic oscillators can be insufficient to quantitatively model and simulate the plasmonic effects. In particular, the above-mentioned plasmon-induced reduction in exciton binding in metallized perovskite cells is impossible to describe with the classical approach to plasmons and needs a quantum method of the Fermi golden rule type applied to the coupling of local plasmons with band electrons in the surrounding semiconductor (perovskite). This coupling concerns plasmon dipole radiation in the near-field zone [18,19], which is not translationally invariant and does not conserve the momentum of an electron during its excitation from the valence band to the conduction band in perovskite. The excitons created by plasmons are thus not diagonal in momentum, and the greater the difference in momenta between the initial (hole) and final (electron) states at electron hopping induced by plasmons, the lower the binding energies of excitons. This is in contrast to an ordinary photo-effect, for which only a diagonal in momentum e-h pairs is created (this is frequently called vertical optical interband electron transition in a semiconductor). The binding of nondiagonal excitons is weaker, which is clear because the oppositely oriented momenta of the hole and the electron tend to decouple the pair. This is, however, a purely quantum effect, beyond the reach of the classical electrodynamic model of plasmons.

In the present paper, we exemplify various problems related to plasmons (usable in photovoltaics), which can be confusing to interpret which can be misinterpreted within the classical approach with the classical approach, as it frequently relies upon numerical methods for the solutions of Maxwell–Fresnel boundary equations using finite element methods (using popular software packages, e.g., Comsol), and the models of plasmons that are assumed as prerequisites are too simplified.

Nanoparticles of noble metals (such as Au, Ag and Cu) exhibit pronounced surface plasmon resonance in the range of the visible light spectrum and therefore are suitable to mediate the absorption of photons in metallized solar cells operating mostly within the visible part of the sunlight spectrum. It has been demonstrated both experimentally and theoretically that metallic nanoparticles deposited on top of conventional p-n junction solar cells (e.g., Si-based cells or CIGS) cause a significant increase in cell efficiency due to the coupling of surface dipole plasmons in the near-field zone in metallic components to band electrons in semiconductor substrates (cf. for a review of experiments and theory, see [12]). Simultaneously, it has been shown that the strengthening of the electric field of the incident light wave near the curvature of metallic nanoparticles (an effect that is accounted for by the solution of the Maxwell–Fresnel boundary problem in conventional classical simulations using the finite method numerical solution of electrodynamic differential equations) provides only 5% of the efficiency increase, whereas 95% of the plasmonic photovoltaic effect is caused by the quantum coupling of plasmon dipoles in the near-field zone with electrons from the semiconductor substrate, accounted for by the Fermi golden rule [20]. The inclusion of this quantum coupling is thus decisive in the explanation of the experimentally observed plasmon photovoltaic effect: it is not of the geometry type (classical Maxwell–Fresnel problem) but is related to the increase in the damping of plasmons, which is impossible to account for by classical modeling with the dielectric function of the Drude type [21] with the optical data measured in the bulk. In the case of chemical solar cells, the reduction in exciton binding energy by plasmons is completely beyond the reach of classical electrodynamics but is possible to assess by applying the Fermi golden rule.

In the present paper, we will demonstrate how bulk optical data for the dielectric function for classical simulations can differ from the actual optical parameters in the case of the nanoscale confinement of a metal. This is particularly clear in the case of plasmon oscillations in metallic nanoparticles, in which anharmonic oscillations occur with large discrepancies in both the frequency and damping rate in comparison to the conventional damped harmonic oscillator.

## 2. Microscopic Description of Plasmons in a Metallic Nanoparticle

Plasmons have been studied since the beginning of the XX century in the framework of classical Maxwell electrodynamics based on the formulation of the Fresnel boundary problem between a dielectric and metal [18,19,21]. For the case of a spherical or spheroidal shape of this interphase border, analytical solutions have been found [22,23], provided that the dielectric function of the metal, including the volume bulk plasmon, is in the Drude form [21],

(1) $$\varepsilon \left(\omega \right)=1-\frac{\omega_{p}^{2}}{\omega^{2}+i\gamma \omega }=1-\frac{\omega_{p}^{2}}{\omega^{2}+\gamma^{2}}+i\frac{\gamma \omega_{p}^{2}}{\omega (\omega^{2}+\gamma^{2})},$$

where $\omega_{p}=\sqrt{\frac{e^{2}n}{\varepsilon_{0}m}}$ (in SI, in Gauss units $\sqrt{\frac{4\pi e^{2}n}{m}}$) is the volume bulk plasmon frequency in the framework of the oscillatory model of the dielectric function described by Lorentz [21] (*e* is the electron charge, *m* is the electron mass, n=N/V is the concentration of free electrons in the metal, and $\varepsilon_{0}$ is the dielectric constant). γ defines the damping of plasmons in the bulk metal caused by electron scattering on crystalline perturbations—admixtures, lattice defects, phonons and other electrons—and is corrected for the nanoparticle geometry by the scattering of electrons on the boundary of a particle [24,25,26,27],

(2) $$\gamma =\frac{1}{\tau_{0}}=\frac{v_{F}}{2\lambda_{b}}+\frac{Cv_{F}}{2a},$$

where *a* is the nanoparticle radius, *C* is the constant of unity order to account for the type of electron scattering on the nanoparticle boundary, $v_{F}$ is the Fermi velocity of electrons, and $\lambda_{b}$ is the mean free path of electrons in the bulk metal.

The plasmon damping modeled by (2) is, however, underestimated, both in the case of plasmon oscillations in metallic nanoparticles in dielectric surroundings and if plasmons in a metallic nanoparticle are coupled in the near-field zone with some absorbing medium, such as the semiconductor substrate on which metallic components are deposited (e.g., for metallized Si or CIGS solar cells [1,2,3,4,5,6,7,8,9,10]) or in which they are fully embedded (e.g., for metallized perovskite cells [13,14,15]).

To microscopically describe plasmons, we adopt the random-phase approximation (RPA) model developed by Pines and Bohm [28] in 1952 to derive the frequency of bulk plasmons. Using the second-order time derivative of the operator of the local density of electrons in the Heisenberg picture, the oscillating mode of this density has been identified from the term related to electron repulsion [28]. It is somewhat surprising that plasmons in bulk metal exist due to the repulsion of electrons and not due to attraction to positive jellium. This means that the coherence in the oscillations of the charge density requires the mutual interaction of electrons; otherwise, such oscillations cannot be organized. The generalization of the quantum RPA method for a metallic nanoparticle was then performed [29]. The Heisenberg equation for local charge dynamics in a finite metallic particle gains additional structure because of the sharp rim of jellium (absent in bulk metal), which produces, due to gradients in the Hamiltonian, some Dirac delta terms on the jellium rim in the dynamic Heisenberg equation and a related new class of plasmon excitations called surface plasmons. Thus, in nanoparticles, there occur two families of plasmons instead of a sole mode in the bulk [29], related to Dirac delta terms in a dynamic equation and those without any singularity.

The latter family consists of volume plasmons of the form for a local charge density fluctuation δρ(r) in a spherical nanoparticle [29],

(3) $$\delta \rho \left(r\right)=n\sum_{l=1}^{\infty }\sum_{m=-l}^{l}\sum_{i=1}^{\infty }A_{lmi}j_{l}\left(k_{li}r\right)Y_{lm}\left(\Omega \right)sin\left(\omega_{li}t\right),\;\mathrm{for}\;r<a,$$

where *n* is the uniform equilibrium density of electrons, $j_{l}\left(\xi \right)=\sqrt{\frac{\pi }{2\xi }}I_{l+1/2}\left(\xi \right)$ is the spherical Bessel function, $Y_{lm}\left(\Omega \right)$ is the spherical function, Ω is the conventional notation for spherical angles, $\omega_{li}=\omega_{p}\sqrt{1+\frac{x_{li}^{2}}{k_{TF}^{2}a^{2}}}$ represents the frequencies of volume plasmon self-oscillations, $k_{TF}=\sqrt{\frac{6ne^{2}}{\epsilon_{F}}}$ is the Thomas–Fermi inverse length ($\epsilon_{F}$ is Fermi energy), and $x_{li}$ represents the nodes of the Bessel function $j_{l}\left(\xi \right)$, with i=1,2,3…, $k_{li}=x_{li}/a$. We see that the self-frequencies of these volume-type plasmon (for r<a) oscillations in a metallic nanoparticle are $\omega_{li}>\omega_{p}$. The confinement increases the energy of volume-type oscillations of the electron density.

The former class of plasmons consists of oscillations on the surface only (those related to the Dirac delta on the jellium rim): these are the surface plasmons,

(4) $$\delta \rho \left(r\right)=\sum_{l=1}^{\infty }\sum_{m=-l}^{l}\frac{B_{lm}}{a^{2}}Y_{lm}\left(\Omega \right)sin\left(\omega_{l0}t\right)\delta \left(r-a\right),$$

where $\omega_{l0}=\omega_{p}\sqrt{\frac{l}{2l+1}}$ denotes the frequencies of surface electron self-oscillations; note that $\omega_{l0}<\omega_{p}$. The detailed derivation of (3) and (4) is presented in [12].

### 2.1. Excitation of Surface Plasmons

To illustrate the above-listed variety of plasmonic modes in a nanoparticle, let us consider a metallic nanoparticle with the radius a∈(5,100) nm located in a dielectric medium with permittivity ε and exposed to the plane wave electro-magnetic field with the frequency ω (the field of incident photons). The surface plasmon resonances $\omega_{l0}=\omega_{p}\sqrt{\frac{l}{2l+1}}$ of the multipole type (dipole type for l=1, quadrupole type for l=2, and so on) have increasing frequencies with increasing *l* (but the rate of this growth gradually diminishes as *l* increases). The smallest frequency, the dipole type for $\omega_{1}=\omega_{p}\frac{1}{\sqrt{3}}$ (the second index 0 is suppressed here in $\omega_{l0}$, as it had been introduced to fit with the volume plasmon spectrum $\omega_{li}=\omega_{p}\sqrt{1+\frac{x_{li}^{2}}{k_{TF}^{2}a^{2}}}$, for which *i* is the number of zeros of the Bessel function on the particle rim, and it attains values of 1,2,3,…), changes in various materials according to $\omega_{p}=\sqrt{\frac{4\pi e^{2}n}{m}}$ and $k_{TF}$ via the free electron concentration *n*. In metals, this concentration varies with the density and the valency, but it is typically of the order of the Avogadro number per 1 cm^3^. Thus, the photon wavelength corresponding to $\omega_{1}$ in Au, Ag or Cu is of the order of 500 nm, much larger than the size of the considered nanoparticles. Hence, photons with a resonant frequency $\omega_{1}$ have an almost uniform electric field distribution over the whole nanoparticle and can excite only the dipole mode of surface plasmons. The excitation of a quadrupole mode requires larger nanoparticles (a>∼75 nm for Au).

### 2.2. Dipole-Type Surface Plasmon

In medium-sized metallic nanoparticles, the dipole mode l=1 of surface plasmons can only be excited by photons (as described above, due to the space homogeneity of the resonant e-m wave along the whole particle). The fluctuation of the charge density can thus be represented as (acc. to Equation (4))

(5) $$\delta \rho \left(r,t\right)=\left\{\begin{array}{c} 0,\;\;r<a,\\ \sum_{m=-1}^{1}Q_{1m}\left(t\right)Y_{1m}\left(\Omega \right)\delta \left(a-r+\epsilon \right)\;r\geq a,\;\epsilon =0+, \end{array}\right.$$

The limiting approach to the surface r≥a,r→a, is taken for mathematical rigor reasons to properly define the Dirac delta inside some open set, cf. [29]. The l=1 surface plasmon oscillations can thus be represented by an oscillating dipole fixed to the nanoparticle center,

(6) $$d\left(t\right)=e\int d^{3}rr\delta \rho \left(r,t\right)=\frac{\sqrt{2\pi }}{\sqrt{3}}ea^{3}\left[Q_{1,1}\left(t\right),Q_{1,-1}\left(t\right),\sqrt{2}Q_{1,0}\left(t\right)\right]$$

which satisfies the RPA dynamic equation [12,29],

(7) $$\left[\frac{\partial^{2}}{\partial t^{2}}+\frac{2}{\tau_{0}}\frac{\partial }{\partial t}+\omega_{1}^{2}\right]d\left(t\right)=\frac{a^{3}4\pi e^{2}n_{e}}{3m}E\left(t\right)=\varepsilon a^{3}\omega_{1}^{2}E\left(t\right).$$

where E(t) is the time-oscillating homogeneous equation for where E(t) is the time-oscillating, homogeneous over the whole particle, electric field of incident e-m radiation. the whole particle electric field of incident e-m radiation. Equation (7) is of the harmonic oscillator type (damped and forced). Note that this equation is nontrivial, as the frequency $\omega_{1}$ originates from the electron repulsion needed to synchronize the oscillations of all electrons, similar to $\omega_{p}$ identified in the bulk using the RPA method by Pines and Bohm [28,30]. This equation for a finite nanoparticle must, however, include an additional term related to the damping of plasmons due to the Lorentz friction of an oscillating dipole [18,19], which is absent in the bulk. Oscillating charges themselves emit an e-m wave, which absorbs the energy of plasmons and eventually quenches the oscillations. The effect is small for a single electron but grows proportionally to the number of electrons participating in the collective synchronized oscillations, as in the case for plasmons. In ultra-small clusters of metals, the total number of electrons is still small, and the Lorentz friction is negligible for a<5 nm. However, with an increase in *a*, the number of electrons grows with $a^{3}$ and quickly attains a large value, so the role of the Lorentz friction starts to be important. The Lorentz friction must be added to the dynamic equation (Equation (7)) as an additional electric field term $E_{L}=\frac{2}{3c^{3}}\frac{\partial^{3}d(t)}{\partial t^{3}}$ [18,19] on the right-hand side of the equation, so the dynamic equation attains the form

(8) $$\left[\frac{\partial^{2}}{\partial t^{2}}+\frac{2}{\tau_{0}}\frac{\partial }{\partial t}+\omega_{1}^{2}\right]d\left(t\right)=\varepsilon a^{3}\omega_{1}^{2}E\left(t\right)+\varepsilon a^{3}\omega_{1}^{2}E_{L},$$

or, in a more explicit form, limited to the case when E=0 (the homogeneous differential equation for self-frequencies),

(9) $$\left[\frac{\partial^{2}}{\partial t^{2}}+\omega_{1}^{2}\right]d\left(t\right)=\frac{\partial }{\partial t}\left[-\frac{2}{\tau_{0}}d\left(t\right)+\frac{2}{3\omega_{1}\sqrt{\varepsilon }}{\left(\frac{\omega_{p}a}{c\sqrt{3}}\right)}^{3}\frac{\partial^{2}}{\partial t^{2}}d\left(t\right)\right].$$

Because of the third-order time derivative of the dipole in the Lorentz friction term, the dynamic equation is not of the harmonic oscillatory type. It is still a linear differential equation but of the third order: its solution will still describe oscillations, but they are different from those for the harmonic oscillator second-order differential equation.

## 3. Anharmonicity of Dipole Surface Plasmons

To assess the deviation from harmonicity due to the Lorentz friction for plasmons, we must solve Equation (9). This can be completed analytically by using the Fourier transform, i.e., assuming $d\left(t\right)∼e^{i\Omega t}$. The third-order differential equation has three (complex in general) solutions for Ω,

(10) $$\begin{array}{cc} \Omega_{1}= & -\frac{i}{3l}-\frac{i2^{1/3}\left(1+6lq\right)}{3lA}-\frac{iA}{2^{1/3}3l}=i\alpha \in Im,\\ \Omega_{2}= & -\frac{i}{3l}+\frac{i\left(1+i\sqrt{3}\right)\left(1+6lq\right)}{2^{2/3}3lA}+\frac{i(1-i\sqrt{3})A}{2^{1/3}6l}=\omega +i\frac{1}{\tau },\\ \Omega_{3}= & -\omega +i\frac{1}{\tau }=-\Omega_{2}^{*}, \end{array}$$

where $A={\left(2+27l^{2}+18lq+\sqrt{4{\left(-1-6lq\right)}^{3}+{\left(2+27l^{2}+18lq\right)}^{2}}\right)}^{1/3}$, $q=\frac{1}{\tau_{0}\omega_{1}}$ and $l=\frac{2}{3\sqrt{\varepsilon }}{\left(\frac{a\omega_{p}}{c\sqrt{3}}\right)}^{3}$.

We see that the solutions given by $\Omega_{2}$ and $\Omega_{3}$ are of the oscillating type with damping ($i\Omega_{2}$ and $i\Omega_{3}$ are mutually conjugated, and thus, $\Omega_{2}$ and $\Omega_{3}$ have real parts with the opposite sign, whereas for the same imaginary parts, the latter is positive, displaying the damping rate). The solution $\Omega_{1}$ (pure imaginary) is an unstable and exponentially rising solution (for a negative sign of α). This unstable solution is the well-known artifact in Maxwell electrodynamics [19], which corresponds to the infinite self-acceleration of the free charge by the Lorentz friction force—the solution of the equation $m\dot{v}=const.\ddot{v}$. This artifact is associated with the formal renormalization of the field mass of the charge—infinite for point-like charge and canceled in an artificial manner by an arbitrary assumed negative infinite non-field mass, resulting in the ordinary mass of, e.g., an electron, which is, however, not properly defined mathematically. The unphysical singular solution $\Omega_{1}$ must be discarded. The other oscillatory-type solution $\Omega_{2}$ (or the $\Omega_{3}$ equivalent via conjugation) resembles the solution of the ordinary damped harmonic oscillator, though with a different attenuation rate and frequency and a different mutual relation between ω and τ. The harmonic type for this relation, $\omega =\omega_{1}\sqrt{1-\frac{1}{{\left(\tau \omega_{1}\right)}^{2}}}$, is not maintained here.

**Figure 1:**
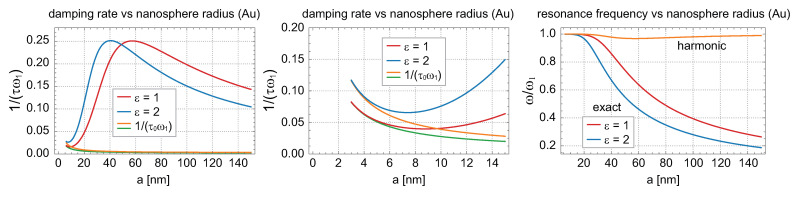
Imaginary and real parts of the solution of Equation (9), i.e., the damping rate and the self-frequency of the dipole mode of surface plasmon resonance in a metallic nanoparticle (Au), given by Equation (10), versus the radius of the nanosphere *a* ($\frac{1}{\tau }$ and ω in units of $\omega_{1}$). The resonances in a vacuum (ε=1) and in dielectric surroundings with ε=2 are compared. The decrease in $\frac{1}{\tau_{0}}$ with *a* acc. to Equation (2) is also plotted in the left panel (zoom of the cross-over region from the left is shown in the central panel). In the right panel, the frequency for the harmonic damped oscillator, $\omega =\omega_{1}\sqrt{1-\frac{1}{{\left(\tau \omega_{1}\right)}^{2}}}$, is also plotted (upper curve) for comparison with the exact anharmonic solution.

The functions ω and $\frac{1}{\tau }$ given by Equation (10) (in dimensionless units, i.e., divided by $\omega_{1}$) are plotted in Figure 1 versus the nanosphere radius *a* for illustration. The deviation from the harmonic damping oscillator dependence $\omega =\omega_{1}\sqrt{1-\frac{1}{{\left(\tau \omega_{1}\right)}^{2}}}$ is very large and visualized in Figure 1 (right panel).

In Figure 1 (left panel) (and from the solution in (10)), we see that the scattering of electrons ($\frac{1}{\tau_{0}}$, which decreases with *a* acc. to Equation (2)) is important for plasmon damping for a<10 nm, whereas, for larger nanoparticles, the damping of the dipole surface plasmon is dominated by the extremely high Lorentz friction: it attains its maximum at ca. a≃57 nm (Au in a vacuum) and decreases again to the small value in the bulk. At its maximum, the damping due to the Lorentz friction is two orders of magnitude greater than that caused by electron scattering (for data from Table 1).

Therefore, if one takes the Drude-type dielectric function within the Lorentz harmonic oscillatory model (1) with $\gamma =\frac{1}{\tau_{0}}$ in the numerical classical simulation of plasmon resonance (by solving Maxwell equations with predefined oscillatory-type dielectric functions), the error can be induced by neglecting the Lorentz friction for nanoparticles with a radius larger than ca. 10 nm (the largest is around a∼57 nm for Au at ε=1, though vanishing in the bulk). Plasmons are not self-consistent solutions of Maxwell equations but are conditioned by the inter-electron repulsion, not included explicitly in the Maxwell–Fresnel problem, which makes room for phenomenological modeling, which must be, however, performed with care (as a metallic nanoparticle is a different plasmonic material from the bulk metal).

## 4. Anharmonicity for Plasmon-Polaritons

In the case of synchronized surface plasmon oscillations in the periodic linear alignment of metallic nanoparticles (nano-chains), strong radiation due to Lorentz friction in particular segments of the chain completely disappears. This astonishing phenomenon is caused by the coupling of dipoles oscillating on chain segments in a synchronized manner, resulting in the wave-type propagation of a plasmon-polariton [31,32,33,34,35,36]. The interaction of dipoles in consecutive segments of the chain can be expressed by the electric field created around the metallic nanoparticle with an oscillating dipole of surface plasmons (the magnetic field does not contribute significantly to this interaction) [18,19],

(11) $$\begin{array}{c} E\left(r,r_{0},t\right)=\frac{1}{\varepsilon }\left(-\frac{\partial^{2}}{v^{2}\partial t^{2}}\frac{1}{r_{0}}-\frac{\partial }{v\partial t}\frac{1}{r_{0}^{2}}-\frac{1}{r_{0}^{3}}\right)D\left(r,t-r_{0}/v\right)\\ +\frac{1}{\varepsilon }\left(\frac{\partial^{2}}{v^{2}\partial t^{2}}\frac{1}{r_{0}}+\frac{\partial }{v\partial t}\frac{3}{r_{0}^{2}}+\frac{3}{r_{0}^{3}}\right)n_{0}\left(n_{0}\cdot D\left(r,t-r_{0}/v\right)\right), \end{array}$$

where $r_{0}$ is the vector linking r—the position of the dipole center—to an arbitrary point outside the nanosphere; $n_{0}=\frac{r_{0}}{r_{0}}$; $v=\frac{c}{\sqrt{\varepsilon }}$ is the light velocity in the surrounding medium; and $D\left(r,t-r_{0}/v\right)=D_{0}\left(r\right)e^{i\omega (t-r_{0}/v)}$ is the dipole of the surface plasmon located on the nanoparticle with a center in r (including relativistic retardation). The terms with denominators of $r_{0}^{3}$, $r_{0}^{2}$ and $r_{0}$ are usually referred to as the near-field, medium-field and far-field components of the oscillating dipole field, respectively.

Using (11), one can extend the dynamic equation (Equation (8)) for dipole oscillations in the metallic nanosphere, including impacts from all other segments in the chain [12]; the segments can be numbered by consecutive integers *l*, and the span between neighboring segments is denoted by *d*. Hence, dipole oscillations on the *l*-th sphere must satisfy the equation

(12) $$\begin{array}{c} \left[\frac{\partial^{2}}{\partial t^{2}}+\frac{2}{\tau_{0}}\frac{\partial }{\partial t}+\omega_{1}^{2}\right]D_{\alpha }\left(ld,t\right)\\ =\varepsilon \omega_{1}^{2}a^{3}\sum_{m=-\infty ,\;m\neq l}^{m=\infty }E_{\alpha }\left(md,t-\frac{|l-m|d}{v}\right)+\varepsilon \omega_{1}^{2}a^{3}E_{L\alpha }\left(ld,t\right)+\varepsilon \omega_{1}^{2}a^{3}E_{\alpha }\left(ld,t\right), \end{array}$$

where α=z denotes the longitudinal polarization of dipole oscillations with respect to the chain orientation (in the *z*-direction), and α=x(y) is the transverse polarization. The first term on the r.h.s. of Equation (12) describes the dipole-type coupling between nanospheres, and the other two terms correspond to the contributions to plasmon attenuation due to the Lorentz friction and the forcing field due to an external electric field. According to Equation (11), we have

(13) $$\begin{array}{c} E_{z}\left(md,t\right)=\frac{2}{\varepsilon d^{3}}\left(\frac{1}{{\left|m-l\right|}^{3}}+\frac{d}{{v|m-l|}^{2}}\frac{\partial }{\partial t}\right)D_{z}\left(md,t-|m-l|d/v\right)\\ E_{x(y)}\left(md,t\right)=-\frac{1}{\varepsilon d^{3}}\left(\frac{1}{{\left|m-l\right|}^{3}}+\frac{d}{{v|m-l|}^{2}}\frac{\partial }{\partial t}+\frac{d^{2}}{v^{2}\left|m-l\right|}\frac{\partial^{2}}{\partial t^{2}}\right)D_{x(y)}\left(md,t-|m-l|d/v\right). \end{array}$$

Due to periodicity, one can propose the wave-type solution

(14) $$\begin{array}{c} D_{\alpha }\left(ld,t\right)=D_{\alpha }\left(k,t\right)e^{-ikld},\\ 0\leq k\leq \frac{2\pi }{d}. \end{array}$$

Thus, the Fourier picture of Equation (12) related to the exponent in (14) attains the form

(15) $$\begin{array}{c} \left(-\omega^{2}-i\frac{2}{\tau_{0}}\omega +\omega_{1}^{2}\right)D_{\alpha }\left(k,\omega \right)\\ =\omega_{1}^{2}\frac{a^{3}}{d^{3}}F_{\alpha }\left(k,\omega \right)D_{\alpha }\left(k,\omega \right)+\varepsilon a^{3}\omega_{1}^{2}E_{0\alpha }\left(k,\omega \right), \end{array}$$

with:

(16) $$\begin{array}{c} F_{z}\left(k,\omega \right)=4\sum_{m=1}^{\infty }\left(\frac{cos(mkd)}{m^{3}}cos\left(m\omega d/v\right)+\omega d/v\frac{cos(mkd)}{m^{2}}sin\left(m\omega d/v\right)\right)\\ +2i\left[\frac{1}{3}{\left(\omega d/v\right)}^{3}+2\sum_{m=1}^{\infty }\left(\frac{cos(mkd)}{m^{3}}sin\left(m\omega d/v\right)\right.\right.\\ \left.\left.-\omega d/v\frac{cos(mkd)}{m^{2}}cos\left(m\omega d/v\right)\right)\right],\\ F_{x(y)}\left(k,\omega \right)=-2\sum_{m=1}^{\infty }\left(\frac{cos(mkd)}{m^{3}}cos\left(m\omega d/v\right)+\omega d/v\frac{cos(mkd)}{m^{2}}sin\left(m\omega d/v\right)\right.\\ \left.-{\left(\omega d/v\right)}^{2}\frac{cos(mkd)}{m}cos\left(m\omega d/v\right)\right)\\ -i\left[-\frac{2}{3}{\left(\omega d/v\right)}^{3}+2\sum_{m=1}^{\infty }\left(\frac{cos(mkd)}{m^{3}}sin\left(m\omega d/v\right)+\omega d/v\frac{cos(mkd)}{m^{2}}cos\left(m\omega d/v\right)\right.\right.\\ \left.\left.-{\left(\omega d/v\right)}^{2}\frac{cos(mkd)}{m}sin\left(m\omega d/v\right)\right)\right]. \end{array}$$

The Lorentz friction contributes to the imaginary parts of $F_{z}$ and $F_{x(y)}$ (the terms $∼i\frac{2}{3}{\left(\omega d/v\right)}^{3}$). The argument ω for each *k* (complex, in general) will define the frequency of the plasmon-polariton $Re\omega_{k}$ and its damping $Im\omega_{k}$. $ImF_{z}$ (and $ImF_{x(y)}$) will define radiation losses beyond the scattering damping $\frac{1}{\tau_{0}}$. One can calculate these imaginary parts by direct analytical summations [37] (using trigonometric relations $sin\alpha +sin\beta =2sin\frac{\alpha +\beta }{2}cos\frac{\alpha -\beta }{2}$ and $cos\alpha +cos\beta =2cos\frac{\alpha +\beta }{2}cos\frac{\alpha -\beta }{2}$ in (16)),

(17) $$\left\{\begin{array}{c} \sum_{m=1}^{\infty }\frac{sin(mz)}{m}=\frac{\pi -z}{2},\;\;\mathrm{for}\;\;0<z<2\pi ,\\ \sum_{m=1}^{\infty }\frac{cos(mz)}{m^{2}}=\frac{\pi^{2}}{6}-\frac{\pi }{2}z+\frac{1}{4}z^{2},\;\;\mathrm{for}\;\;0<z<2\pi ,\\ \sum_{m=1}^{\infty }\frac{sin(mz)}{m^{3}}=\frac{\pi^{2}}{6}z-\frac{\pi }{4}z^{2}+\frac{1}{12}z^{3},\;\;\mathrm{for}\;\;0<z<2\pi . \end{array}\right.$$

This leads to a surprising result,

(18) $$ImF_{z}\left(k,\omega \right)\equiv 0\;\mathrm{and}\;ImF_{x(y)}\equiv 0,\;\mathrm{for}\;0<kd\pm \omega d/v<2\pi ,$$

which means that the plasmon-polariton does not radiate any energy in the chain, despite each of its segments separately exhibiting extremely high Lorentz friction losses. In the chain, the ransfer In the chain, the gain of energy due to dipole coupling of energy due to dipole coupling between all segments completely balances the individual Lorentz friction losses. This effect is inaccessible to numerical simulations of the classical electrodynamic response for a chain, unless the anharmonic Lorentz friction term is accurately included. The absence of the e-m signature of the plasmon-polariton is the pronounced manifestation of the anharmonicity of plasmon oscillations. The property in (18) can serve as the exact sum rule for the verification of numerical simulations of plasmon-polariton kinetics.

The possible application of synchronized plasmon-polaritons in periodic arrays of metallic nanostructures in photovoltaics has not been explored as of yet, but because of the qualitative change in the radiation properties of plasmons in separated nanoparticles in comparison to periodic arrays with w plasmon-polaritons in the metallic nanostructures, it may offer new scenarios for the better harvesting of sunlight. The wave-type guidance of plasmon-polaritons in metallic nanochains, almost perfect, without radiation losses, may serve to transport energy to the inside of a photovoltaic structure, increasing the range of the plasmonic photovoltaic effect to an arbitrary size. A problem may be encountered, however, in the manufacturing of appropriate nanoparticle chains embedded inside photovoltaic cells, but we suggest some experimental trials (perhaps easier in perovskite cells, where metallic components can be admixed with liquid chemical precursors of solid layers). Note that, presented above, perfect wave-guide plasmon-polariton properties are maintained even for very short chains (of ca. 10–20 nanoparticles because of the very quick convergence of the series in Equation (17)).

## 5. Anharmonicity of Plasmons in Metallic Nanoparticles If They Are Coupled with Nearby Absorption Medium

In the case of metallized solar cells, surface plasmons in metallic nanoparticles couple in their near-field zone of radiation with the band electrons of substrate semiconductors. This strong coupling significantly modifies the dielectric functions of both the metallic components and the semiconductor substrate. They are not the same as those for separated, uncoupled subsystems. To account for this coupling quantitatively, one must utilize the Fermi golden rule, because this effect is purely quantum and cannot be properly described with conventional macroscopic plasmonics (via a solution of Maxwell equations for the multicomponent system). The Fermi golden rule, in this case, takes the form for the probability *w* (per time unit) of the interband electron transition in the semiconductor induced by the perturbation due to surface plasmons in metallic nanoparticles deposited on top of the substrate semiconductor:

(19) $$w\left(k_{1},k_{2}\right)=\frac{2\pi }{\hbar }{\left|<k_{1}\left|w^{+}\right|k_{2}>\right|}^{2}\delta \left(E\left(k_{1}\right)-E\left(k_{2}\right)+\hbar \omega \right),$$

where we assume that Bloch states in the conduction and valence bands are planar waves (for the sake of simplicity),

(20) $$\begin{array}{c} \Psi_{k_{1}}=\frac{1}{{\left(2\pi \right)}^{3/2}}e^{ik_{1}\cdot R-iE\left(k_{1}\right)t/\hbar },\Psi_{k_{2}}=\frac{1}{{\left(2\pi \right)}^{3/2}}e^{ik_{2}\cdot R-iE\left(k_{2}\right)t/\hbar },\\ E\left(k_{1}\right)=-\frac{\hbar^{2}k_{1}^{2}}{2m_{p}^{*}}-E_{g},E\left(k_{2}\right)=\frac{\hbar^{2}k_{2}^{2}}{2m_{n}^{*}}, \end{array}$$

(the indices *n* and *p* refer to electrons from the conduction and valence bands, respectively, and $E_{g}$ is the forbidden gap). The plasmon-induced perturbation is caused by the near-field part of the dipole plasmon electric field (11), which, conserving only terms with $r_{0}^{3}$ denominators, attains the form $E_{\omega }=\frac{1}{\varepsilon r_{0}^{3}}\left[3n_{0}\left(n_{0}\cdot D_{0}\right)-D_{0}\right]$ (for the near-field zone, one can neglect the time retardation, and the Fourier picture of (11) has been taken). For such an electric field, one can find the potential, which enters the Hamiltonian of electrons in a semiconductor, $w^{+}=\frac{e}{\varepsilon r_{0}^{2}}n_{0}\cdot D_{0}$, such that $-\nabla_{r_{0}}w^{+}=eE_{\omega }$ (*e* is the electron charge).

First, one can find (via integration) the matrix element $<k_{1}\left|w^{+}\right|k_{2}>$ for the functions in (20); the analytical calculation [12] gives the result

(21) $$<k_{1}\left|w^{+}\right|k_{2}>=\frac{1}{{\left(2\pi \right)}^{2}}\frac{e}{\varepsilon }\frac{D_{0}\cdot q}{q^{2}}\frac{sinqa}{qa},$$

where $q=k_{2}-k_{1}$.

Next, summation over all initial and final states in both bands must be performed to obtain the total probability of the interband electron excitation, i.e.,

(22) $$\delta w=\int d^{3}k_{1}\int d^{3}k_{2}\left[f_{1}\left(1-f_{2}\right)w\left(k_{1},k_{2}\right)-f_{2}\left(1-f_{1}\right)w\left(k_{2},k_{1}\right)\right],$$

where $f_{1}$ and $f_{2}$ represent the temperature-dependent distribution functions (Fermi–Dirac distribution functions) for the initial and final states, respectively. Emission and absorption are included, but for room temperature, one can assume that $f_{2}≃0$ and $f_{1}≃1$, which gives

(23) $$\delta w=\int d^{3}k_{1}\int d^{3}k_{2}\cdot w\left(k_{1},k_{2}\right).$$

The integration in the above expression gives

(24) $$\begin{array}{c} \delta w=\frac{4}{3}\frac{\mu^{2}\left(m_{n}^{*}+m_{p}^{*}\right)2\left(\hbar \omega -E_{g}\right)e^{2}D_{0}^{2}}{\sqrt{m_{n}^{*}m_{p}^{*}}2\pi \hbar^{5}\varepsilon^{2}}\int_{0}^{1}dx\frac{sin^{2}\left(xa\xi \right)}{{\left(xa\xi \right)}^{2}}\sqrt{1-x^{2}}\\ =\frac{4}{3}\frac{\mu^{2}}{\sqrt{m_{n}^{*}m_{p}^{*}}}\frac{e^{2}D_{0}^{2}}{2\pi \hbar^{3}\varepsilon^{2}}\xi^{2}\int_{0}^{1}dx\frac{sin^{2}\left(xa\xi \right)}{{\left(xa\xi \right)}^{2}}\sqrt{1-x^{2}}, \end{array}$$

where $\mu =\frac{m_{n}^{*}m_{P}^{*}}{m_{n}^{*}+m_{p}^{*}}$ is the reduced effective mass in the semiconductor. In limiting cases, one ultimately obtains

(25) $$\begin{array}{c} \delta w=\left\{\begin{array}{c} \frac{4}{3}\frac{\mu \sqrt{m_{n}^{*}m_{p}^{*}}\left(\hbar \omega -E_{g}\right)e^{2}D_{0}^{2}}{\hbar^{5}\varepsilon^{2}},\;\mathrm{for}\;a\xi \ll 1,\\ \frac{4}{3}\frac{\mu^{3/2}\sqrt{2}\sqrt{\hbar \omega -E_{g}}e^{2}D_{0}^{2}}{a\hbar^{4}\varepsilon^{2}},\;\mathrm{for}\;a\xi \gg 1, \end{array}\right. \end{array}$$

where the parameter $\xi =\frac{\sqrt{2\left(\hbar \omega -E_{g}\right)\left(m_{n}^{*}+m_{p}^{*}\right)}}{\hbar }$ and is related to the semiconductor material parameters. It is easy to notice that for nanoparticles with size a∈(10,100) nm, the lower case holds.

Note that the above formulae are fundamentally distinct from the ordinary photo-effect [38]:

(26) $$\begin{array}{c} \delta w_{0}=\frac{4\sqrt{2}}{3}\frac{\mu^{5/2}e^{2}}{m_{p}^{*2}\omega \varepsilon \hbar^{3}}\left(\frac{\varepsilon E_{0}^{2}V}{8\pi \hbar \omega }\right){\left(\hbar \omega -E_{g}\right)}^{3/2}. \end{array}$$

With the estimation of the probability for the excitation of band electrons in the semiconductor substrate by plasmons from the metallic nanoparticle, one can find the related damping of plasmons and also the correction to the imaginary part of the dielectric function for a semiconductor, as the energy from plasmons is transferred to the semiconductor. This leads to the final form of the plasmon damping term due to coupling with the absorbing semiconductor [12],

(27) $$\frac{1}{\tau^{\prime }\omega_{1}}=\left\{\begin{array}{c} \frac{4\beta \mu \sqrt{m_{n}^{*}m_{p}^{*}}\left(\hbar \omega_{1}-E_{g}\right)e^{2}a^{3}}{3\hbar^{4}\varepsilon },\;\mathrm{for}\;a\xi \ll 1,\\ \frac{4\beta \mu^{3/2}\sqrt{2}\sqrt{\hbar \omega_{1}-E_{g}}e^{2}a^{2}}{3\hbar^{3}\varepsilon },\;\mathrm{for}\;a\xi \gg 1. \end{array}\right.$$

For nanospheres of Au deposited on a Si layer, we obtain

(28) $$\frac{1}{\tau^{\prime }\omega_{1}}=\left\{\begin{array}{c} 44.092\beta {\left(a[nm]\right)}^{3}\frac{\mu }{m}\frac{\sqrt{m_{n}^{*}m_{p}^{*}}}{m},\;\mathrm{for}\;a\ll 0.15\sqrt{m/(m_{n}^{*}+m_{p}^{*})}\;\left[nm\right],\\ 13.648\beta {\left(a[nm]\right)}^{2}{\left(\frac{\mu }{m}\right)}^{3/2},\;\mathrm{for}\;a\gg 0.15\sqrt{m/(m_{n}^{*}+m_{p}^{*})}\;\left[nm\right], \end{array}\right.$$

where for light (heavy) carriers in Si, $m_{n}^{*}=0.19\left(0.98\right)\;m$ and $m_{p}^{*}=0.16\left(0.52\right)\;m$, with *m* being the bare electron mass; $\mu =\frac{m_{n}^{*}m_{p}^{*}}{m_{n}^{*}+m_{p}^{*}}$; and $E_{g}=1.14$ eV, ε=12 and $\hbar \omega_{1}=2.72$ eV ($\beta ∼\frac{h^{2}}{a^{2}}∼10^{-3}$ for a∼50 nm, and *h* is the effective range of the near-field coupling). For these parameters and for nanospheres of radius *a* in the range of 5–50 nm, the second case of Equation (28) applies.

The damping rate given by Equation (27) or Equation (28) greatly exceeds $\frac{1}{\tau_{0}\omega_{1}}$, and it is also larger than the damping of plasmons due to the Lorentz friction in dielectric surroundings. To fit with the experimentally observed efficiency increase in Si-type photodiode setups (as listed in Table 2, taken from [12]), the inclusion of the damping rate (27) is necessary, as demonstrated in [20]. The reason for such a strong increase in the damping of plasmons due to coupling with band electrons in the substrate semiconductor is the allowance of oblique interband transitions of electrons because, for near-field coupling with plasmons, the quasi-momentum of band electrons is not conserved (the matrix element (21) is not diagonal in k, contrary to the ordinary photo-effect [38]). The latter property is the reason for the strong increase in the efficiency of solar cells covered with metallic nanoparticles.

## 6. Conclusions

The deviation from harmonicity in the case of plasmons in metallic nanoparticles is surprisingly large for medium-sized particles. For small particles with a radius a<5 nm, the anharmonic effect is negligible, and the same applies to large particles with a>300 nm. A pronounced anharmonic effect occurs, however, for a∈(10,100) nm (for Au in a vacuum, slightly shifted in response to the permittivity of surroundings ε, and similarly for Ag or Cu). The damping of plasmons related to the Lorentz friction exceeds the scattering losses $\frac{1}{\tau_{0}}$ by two orders of magnitude, and its maximum occurs for a≃57 nm (Au in a vacuum, 38 nm for ε=2). The dependence of the resonant frequency of surface plasmons versus the nanoparticle radius *a* is also significantly different from that for the conventional harmonic oscillator. Hence, the modeling of plasmons with the solution of the classical Maxwell–Fresnel equation (in both the analytic approach, called Mie approximation, and numerical studies using the finite element method for the solution of differential equations, e.g., with commercial systems such as COMSOL) is burdened by a large systematic error if the prerequisite for calculations is the dielectric function for the metal in the Drude form with the optical parameters measured in the bulk. Thus, reliable classical simulations of plasmonic effects, including those addressed by the plasmon-admixture-induced increase in the efficiency of photovoltaic cells, cannot be carried out with the dielectric function for the metal as in the bulk.

The convincing effect caused by the anharmonicity of plasmons is the absence of the e-m signature of plasmon-polaritons in metallic nano-chains. This is an exact result (of the sum rule type) of the perfect balance of Lorentz friction losses with the energy transfer of Lorentz friction losses with the energy gain due to dipole interactions between chain segments, including the near-, medium- and far-field zone radiation of all dipoles, regardless of the size of the nanospheres and their separation in a chain.

The large anharmonic damping of plasmons in metallic nanoparticles plays, however, a very important role. Due to the universal symmetry between absorption and emission for quantum transitions (known, e.g., from optical transitions), one can conclude that strongly emitting plasmons similarly strongly absorb light. This explains, in particular, the experimental observations that even a very sparse layer of metallic nanoparticles (with a surface concentration of 10^8–9^ particles per cm^2^) on a semiconductor can absorb a large part of incident photons and then transfer the energy via a very effective channel to the band electron system in the substrate semiconductor in an ultra-short time, thereby increasing the overall absorption of photons. Such modified materials can serve as both very effective absorbers and emitters of light. We pointed out the large discrepancy between simplified classical assumptions about plasmon oscillations and their true quantum behavior, which is of significance for the reliability of the modeling of plasmonic effects.

## Figures and Tables

**Table 1:** Nanosphere parameters assumed for calculation of damping rates for surface plasmons.

Material	Au	Ag
Bulk plasmon energy, $\hbar \omega_{p}$	8.57 eV	8.56 eV
Bulk plasmon frequency, $\omega_{p}$	1.302 × 10^16^ s^−1^	1.3 × 10^16^ s^−1^
Mie dipole plasmon energy, $\hbar \omega_{1}$	4.94 eV	4.93 eV
Mie frequency, $\omega_{1}=\omega_{p}/\sqrt{3}$	0.752× 10^16^ s^−1^	0.75× 10^16^ s^−1^
Constant in Equation (2), *C*	1.4	1.4
Fermi velocity, $v_{F}$	1.396 × 10^6^ m/s	1.4 × 10^6^ m/s
Bulk mean free path (room temp.), $\lambda_{b}$	53 nm	57 nm

**Table 2:** Examples of the measured values of the photocurrent enhancement in silicon solar cells and silicon photodiodes with deposited metallic nanoparticles.

Metal	Size (nm)	Concentration 1/cm^2^	Enhancement	Ref.
Au	50	$6.6\times 10^{8}$	18%	[1]
Au	100	$9.9\times 10^{8}$	2.8%	[4]
Au	100	$3.2\times 10^{8}$	3.3%	[3]
Au	100	$3.5\times 10^{8}$	3.3%	[2]
Au	∼20	$1.3\times 10^{11}$	20%	[5]
Au	65	$10\times 10^{8}$	18%	[6]
Ag	40	$124\times 10^{8}$	127%	[8]
Ag	12	-	19%	[7]
Al	22:81	40% of surface	21%	[39]

## Data Availability

All data are available within the manuscript.
